# Hard to reach services or survivors? Perceptions of service providers on barriers and facilitators for help-seeking behaviours in addressing domestic violence among British South Asian women

**DOI:** 10.1186/s12889-025-24226-6

**Published:** 2025-09-30

**Authors:** Razia Sultana, Nusrat Husain, Omolade Femi-Ajao, Peter Taylor

**Affiliations:** 1 Division of Psychology and Mental health, University of Manchester, Oxford Road, Manchester, M13 9PL UK; 2 Division of Dentistry, School of Medical Sciences, University of Manchester, Oxford Road, Manchester, M13 9PL UK; 3 University of Manchester, Oxford Road, Manchester, M13 9PL UK

**Keywords:** Domestic violence, Disclosure, Help-seeking, Intimate partner violence, South asian

## Abstract

Domestic violence (DV) against ethnic minority women is often an understudied social and psychological problem in the United Kingdom. The aim of this qualitative research is to fill a gap in the existing literature by identifying service providers’ perceptions about the barriers and facilitators for help-seeking behaviours among British South Asian women who have experienced DV. This study used 18 semi-structured interviews of service providers from third-party organisations. In addition, it used qualitative methods and applied thematic analysis within an ecological framework to analyse interviews with service providers. Five main themes were identified relating to barriers and facilitators for help-seeking behaviours among British South Asian women who have experienced DV. These include: stereotypical thinking and the misuse of religious beliefs; fear of negative consequences; emotional states that act as both barriers and facilitators; informal and formal help-seeking opportunities, as well as the challenges associated with each. These research findings can initiate positive social change by leading to development of culturally appropriate interventions which can bridge the gap between British South Asian women who experience DV. The findings of this study contribute to Sustainable Development Goal (SDG) 5: Gender Equality by identifying key barriers, facilitators, and culturally informed recommendations for help-seeking among British South Asian women experiencing domestic violence. These insights can inform the development of effective policy, practice, and research to address domestic violence as a major public health issue and challenge the intersecting racial, ethnic, and gender inequalities that exacerbate it.

## Introduction

Domestic violence (DV) is a major human rights issue, and one third of women worldwide have suffered from this violence at some point in their lives [1]. DV is most prevalent in the South Asian region [2] and is also reported among South Asian populations in Western countries. DV has also been noted among South Asian populations within Western countries. The Crime Survey for England and Wales 2017 reported that 6.9% of all women aged 16 to 74 had experienced DV, and 3.4% of South Asian women aged 16 to 74 were victims of domestic abuse [3]. The reason for this lower prevalence may be that the national research often uses small and non-representative samples that may underestimate the extent of the problem among South Asian women, or that South Asian women do not seek help and therefore DV is underreported [4]. South Asian domestic abuse cases might be underreported for several reasons, such as fear of the perpetrators, financial insecurity, humiliation related to family honour, and concerns about immigration status—particularly fear of deportation or losing a spousal visa [5–7]. This research has found that lack of support, and unhelpful behaviours among professionals can influence DV survivors in normalising their abuse [8]. Although the statutory services in the UK have already taken the initiative to provide support to DV survivors from minoritised communities, there have also been societal barriers (restrictions on receiving state benefits, systemic racism, refuge provision, poverty, and unemployment) which have discouraged women from leaving abusive relationships and seeking help [9].

In this study, *help-seeking* refers to the process by which individuals reach out for support or assistance in response to domestic violence. This includes *informal help-seeking*, such as turning to family, friends, or community members, and *formal help-seeking*, which involves accessing professional or institutional services such as healthcare, counselling, legal aid, or domestic violence support organisations.

There is a dearth of literature investigating the perceptions of service providers from non-profit/voluntary organisations about DV in the UK, even though they have close relations with survivors and provide diverse services (counselling, legal, medical, housing support, review of safety plans, facilitation of peer support groups, and more). The research that has been conducted has focused on statutory health service providers’ perceptions of DV [10–13]. Some studies have suggested that survivors have a greater willingness to seek help from service providers working for voluntary organisations rather than for statutory services [14, 15] because of the complications of police- and court-related issues [16–18]. This current research collects service providers’ views through interviews regarding their professional training and experience, as well as the nature of their contact with British South Asian (BSA).

The phrase “hard to reach” is frequently used in policy and service design to describe populations that underutilise available support. However, scholars have critiqued this term for implying deficiencies within marginalised communities rather than shortcomings in service accessibility or responsiveness [1]. In the context of domestic violence, this framing risks obscuring the ways in which structural barriers, cultural mismatches, and institutional distrust shape survivor disengagement. This study seeks to explore whether it is indeed the survivors who are hard to reach, or whether the services themselves remain difficult to access—especially for British South Asian women navigating complex cultural and systemic constraints.

While several studies have explored domestic violence among South Asian women, there remains a limited understanding of service providers’ perspectives—particularly from those working in non-profit or voluntary sectors. Furthermore, few studies have applied a qualitative approach to explore in depth the complex cultural, social, and structural barriers to help-seeking among British South Asian women. This study addresses this gap by applying a qualitative research design to gather service providers’ nuanced insights and lived professional experiences. The current qualitative study explores the barriers and facilitators for help-seeking behaviours, specifically among British South Asian (BSA) women, which has not been looked at before now.

In this study, ‘BSA’ refers to members of South Asian citizen groups who were born and brought up in, or have legal permission to live in the UK. A few authors have conducted research, which has identified a number of barriers and facilitators for help-seeking behaviours among South Asian immigrant women, or a combination of both South Asian immigrant and BSA women [14, 19, 20]. The main limitation of these previous studies is that they did not distinguish the problems as per each group (such as differentiating regarding religion, ethnicity, residential status, etc.) and treated all South Asian people as one unit (South Asian). This research identifies differences or similarities in the barriers and facilitators regarding help-seeking behaviours for this BSA women group compared with immigrant women. In addition, several studies have been conducted based on the DV experiences of South Asian ethnic women. This study fills a knowledge gap by researching DV service providers’ perceptions about help-seeking behaviours among BSA women who have experienced DV. In this paper, the focus in on DV perpetrated by partners or husbands. In the UK, people with roots in India, Pakistan, Bangladesh, Nepal and other countries are included in the South Asian ethnic groups [21].

Some of the DV-based research has been conducted using only the ecological model, with the consequence that it offers a limited understanding of gender, religion, ethnic minority women, and racism in the formal support sector [22, 23]. This study uses the adapted ecological intersectional model [24] expanded from Heise’s (1998) ecological model and Crenshaw [23] intersectionality framework. The ecological model was developed to understand the origin of gender-based violence through its four levels (individual, relationship, community, and structural) [25]. Crenshaw [23] used the term “intersectionality” to conceptualise how a person or group’s social, biological or culturally diverse identities (ethnicity, gender, immigration/refugee status, religion, sexual orientation, age, disability, spirituality, language, and education) affect them in terms of a number of discriminations or barriers. In this research, the ecological intersectional model uses the four levels [25] for interpreting the data related to diverse intersectional oppression. In this study, the specific intersectional identarian factors can differentiate BSA women from the other group regarding the barriers and facilitators for help-seeking behaviours related to DV.

The individual level represents a person’s own life experience surrounded by the practice of the cultural norms of victims or perpetrators (e.g. childhood adversity, psychological difficulties). A study of eight South Asian therapists reported that unfavourable forceful cultural upbringing had prevented the disclosure of DV by South Asian women [26]. The relationship level refers to poor connections with families, in-laws, and husbands that have acted to prevent disclosure of abuse. It was found that leaving a marriage is not easy because of the need to protect family honour, and the fear of stigma, shame, and blame [20, 27, 28]. The community level refers to the character and resources available within the local community including negative and positive resources. One study reported that ethnic minority women may seek help from religious leaders and people in the local community after experiencing DV, but do not always receive proper help because of factors including victim-blaming, patriarchal norms and social expectations [29, 30]. The structural level includes factors that might influence the seeking of help for DV including rapid social change, gender, social and economic inequalities, poverty, poor legal protection for victims, cultural norms that support violence, and inequality in the support given by statutory and third-party organisations. Researchers have suggested that minority, refugee and immigrant women maintain traditional gender norms and cultural beliefs that have influenced domestic violence [25, 30–32].

In addition, previous DV-based research has also used the ecological model with immigrant and refugee women, rather than specifically with permanently resident ethnic minority women, born and brought up in developed countries [30, 33, 34]. This integrated ecological intersectional model addresses the research gap by adding the experiences of BSA women as perceived by service providers. The aim of this study is to identify the perception of DV service providers about barriers and facilitators for help-seeking behaviours among BSA women who have experienced DV. This study uses an adapted ecological intersectional model to explore how multiple identity-based factors (e.g., ethnicity, gender, religion) shape help-seeking behaviours among British South Asian women experiencing DV.

## Methods

### Theoretical framework

This study uses an adapted ecological intersectional model, which builds upon Heise’s [25] four-level ecological model and Crenshaw’s (1991) intersectionality framework. The ecological model was developed to understand the origin of gender-based violence through its four levels (individual, relationship, community, and structural) [25], while Crenshaw introduced the concept of intersectionality to examine how overlapping identities—such as ethnicity, gender, and immigration status—shape experiences of discrimination [35].

The adapted ecological intersectional model allows for an integrated analysis of how British South Asian women’s diverse identities interact with systemic, cultural, and relational factors to influence their help-seeking behaviours. This model has been applied here to categorise barriers and facilitators across the four ecological levels while accounting for intersecting forms of oppression.

In this research, the model also aids triangulation by examining whether the perceptions of DV service providers align or contrast with findings from previous survivor-based studies on South Asian women, thus offering a broader picture of help-seeking challenges and opportunities.

### Study design

This research follows a qualitative method, which is particularly appropriate for exploring a sensitive issue such as DV. Qualitative approaches allow for rich, detailed accounts and do not assume prior knowledge of participants’ lived experiences. This method permits participants to express their realities in their own terms and enables a deeper understanding of the barriers and facilitators affecting BSA women’s help-seeking behaviours [36]. This cross-sectional qualitative study using interviews with DV support service providers to investigate their perspectives and views concerning the facilitators and barriers for BSA women when seeking help for DV. The study was approved by the University of Manchester’s University Research Ethics Committee (UREC) (Ref: 2020-7981-13795). A critical realist position is taken in this research, as it assumes that there is a real world out there that is external to people. This perspective suggests that people may share a general understanding of the world, even if their experiences are subjective and context-dependent [37]. This critical realist position combines ontological realism and epistemic relativism [38]. For example, in this current research, data have been collected from DV service providers who have provided support to BSA women experiencing DV; the data analysis and interpretation of findings are considered according to the researcher’s independent observation, knowledge, understanding and experiences. Overall, from the critical realist position, the researcher’s construction of the data and findings may not deliver the original reality. Thematic analysis is applied to analyse the data in order to identify, analyse, and report themes in the data [39].

### Study participants

Service providers from third-party, voluntary, or non-governmental organisations that support women experiencing domestic violence were targeted for recruitment. A purposive sampling strategy was used, and recruitment continued until data saturation was achieved—when no new themes emerged from the interviews.

Eligibility criteria for recruitment included:

- Aged 18 years or above;
- Currently or recently working in a role supporting British South Asian (BSA) women experiencing domestic violence;
- Employed in a third-sector, voluntary, or non-governmental (NGO) organisation in the UK;
- Able to provide informed verbal consent;
- Having direct experience supporting BSA women who are survivors of DV.

There were no exclusion criteria based on ethnicity, as the study aimed to include all service providers who had supported BSA women, regardless of the provider’s own ethnic background.

This research was conducted during the COVID-19 pandemic when the UK Government had declared a ‘lockdown’ in order to maintain social distancing and required all but essential workers to stay at home [40]. The study was therefore undertaken remotely, avoiding in-person contact. DV service providers (18 years old or above) were recruited remotely (online or by mobile phone) from non-profit/voluntary/non-government (NGO)/third-party organisations for this study. Informed consent was obtained from each participant prior to the start of each interview. It was also informed that if any participant was not able to provide consent, they would not be allowed to attend the interview, as informed consent is a crucial part of research ethics and is the permission granted by the participants in full knowledge of the possible consequences, risks and benefits [41]. The recruitment of participants was not limited by ethnic origin, as it was planned to interview all service providers who provided support to British South Asian women with lived experiences of domestic violence, even if they (service providers) did not identify as British South Asian. Participants contacted the organisations targeted remotely and were also recruited via research flyers shared on Twitter, LinkedIn, Facebook, Instagram and other social media and professional media platforms. Full recorded verbal consent was obtained from all participants. The interviews started on 31 st July 2020, and recruitment was completed on 16th February 2021. A total of 18 service provider participants attended. Although the researcher did not ask service providers about their own lived experiences, some of them (service providers) willingly shared that they had had DV experiences in their lifetimes. For this reason, the researcher did not disclose the name of the organisations they worked for to ensure the safety and security of the participants.

### Data collection

A semi-structured interview schedule was adapted from Femi-Ajao [42] to investigate the study’s research question. The adaptation process involved rewording questions to reflect the terminology and context relevant to DV service providers rather than survivors. Items focusing on direct personal experiences of DV were removed, while new questions were added to explore professional training, perceived cultural barriers, and facilitators in supporting BSA women. The revised guide was reviewed by the research team to ensure cultural appropriateness and alignment with the study’s aims. Interviews lasted for 45 to 90 min and were recorded using an encrypted Dictaphone—a secure audio recording device that ensures data protection by encrypting recordings to prevent unauthorised access. Some of the service provider participants had also experienced and survived domestic abuse, and while the focus of this research was not on the lived experience of the service providers, there was an opportunity for them to share their experiences if they wished to do so. Verbal consent was obtained and audio recorded at the beginning of the interview due to the digital nature of the study and to ensure that consent was documented in accordance with ethical guidelines. The researcher created pseudonyms for study participants at the beginning of each interview. This process was used to ensure that any documents containing sensitive data were anonymised from the beginning of the study. Interviews were offered with the following choice of language options: English, Urdu, Hindi and Bengali, and the primary researcher administered the interviews as she is a multilingual speaker. However, all service providers chose to speak in English. Participation in the research was voluntary and the interview times were selected at the convenience of the service providers.

All interview recordings were transcribed verbatim by a professional transcription service (Business Friend). All transcripts were reviewed again by the primary author (RS), and the co-author (OF) to ensure credibility [43]. The transcripts were denaturalised; that is, grammatical errors, fillers, and repeated words were removed to improve clarity, while preserving the core meaning and intent of participants’ responses. The transcripts were anonymous with personal information removed to prevent the identification of participants.

#### Data analysis

**Fig. 1:**
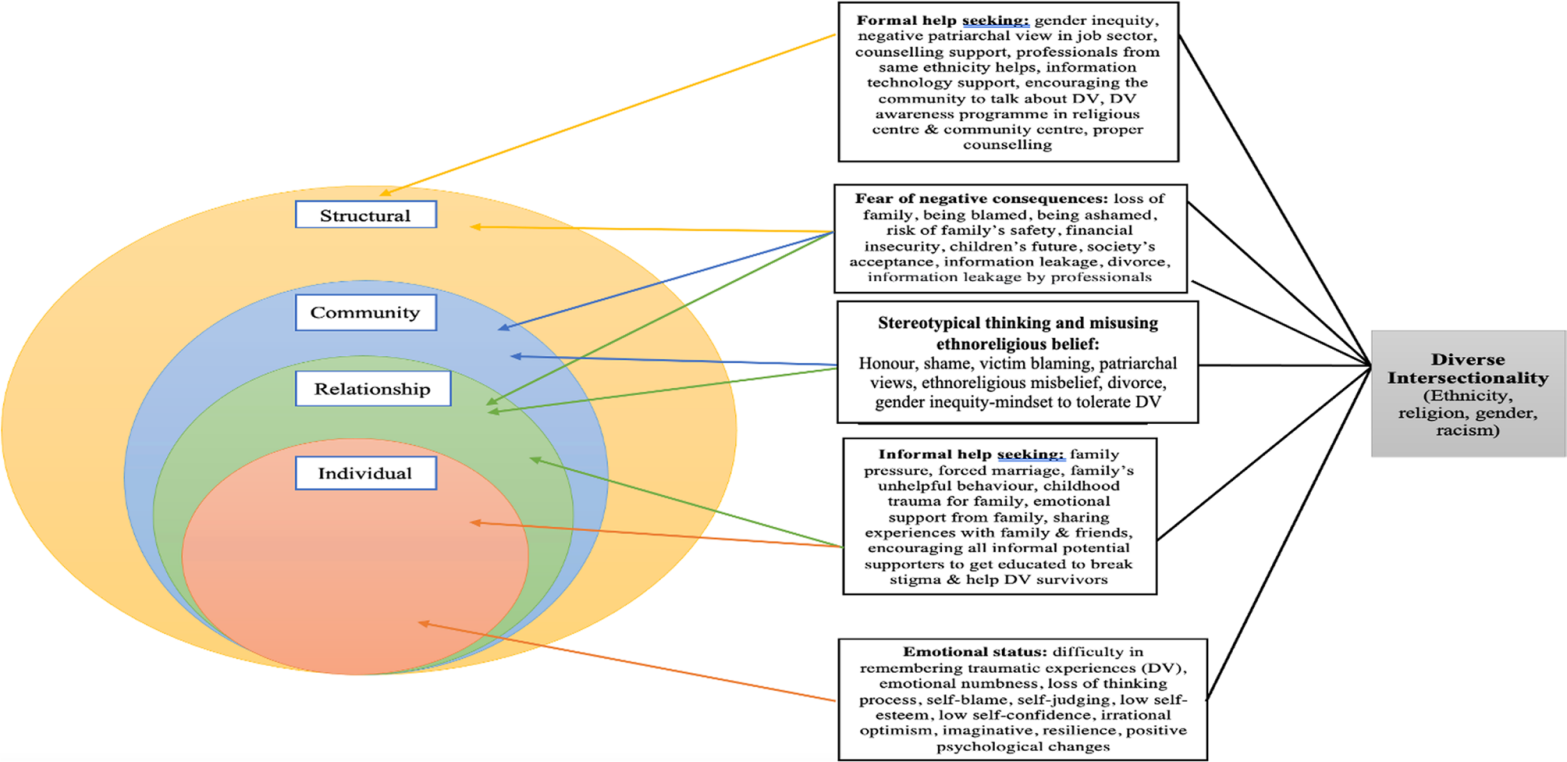
Ecological Intersectionality Model adapted from LaVoi [24]

The ecological intersectional model [24] was used to guide the analysis to identify barriers and facilitators related to DV at each ecological level. The interview transcripts were analysed using the critical realist approach and a hybrid process of deductive and inductive theoretical thematic analysis [42]. The deductive approach provided an initial sound grounding through the ecological intersectional model, and the researcher included an inductive process to allow the realities of others to be clearly represented in the data analysis [45]. From the critical realist perspective, a hybrid process of deductive and inductive thematic analysis was undertaken. In the deductive phase, the literature—particularly the adapted ecological intersectional model—guided the creation of an initial coding framework, helping to identify relevant domains such as individual, relationship, community, and structural barriers. The primary researcher brought to the analysis a perspective shaped by her academic background in clinical psychology and extensive professional experience in DV research and practice within British South Asian communities. While not drawing from personal lived experience as a survivor, the researcher engaged in ongoing reflexive journaling to remain aware of how her cultural understanding, professional insights, and positionality may influence theme development and interpretation. During the inductive phase, the researcher conducted open coding to allow themes to emerge directly from the data. In the final stage, the literature was revisited to refine, label, and contextualise the emergent themes, ensuring both theoretical grounding and fidelity to participants’ lived experiences. In addition to using a deductive approach guided by the adapted ecological intersectional model, an inductive process was also applied to ensure that themes emerged directly from the data. The primary researcher conducted open line-by-line coding of several transcripts without applying any pre-set categories. These initial codes captured participants’ language, meanings, and lived experiences. The codes were then grouped into broader categories that reflected recurring patterns across the dataset. These emerging themes were reviewed by the research team and discussed in relation to the ecological framework, ensuring that inductively derived insights were retained and not forced to fit pre-existing categories. This dual approach allowed participant realities to be represented authentically while benefiting from the organising structure of the theoretical model. From the critical realist perspective, themes were developed from the data and interpreted through the researcher’s academic background in psychology, ongoing reflexive engagement, and understanding of the literature, rather than personal experience [46]. It triangulates among different perspectives to obtain a broader picture of the challenges facing these women and is also helpful in identifying whether or not DV service providers and DV survivors have similar views in comparison with the previous South Asian women survivor-based DV research. South Asian women may suffer DV not only from partners, but also from multiple family members [19]. Service providers’ perceptions and experiences were analysed and organised into overarching codes and themes that reflected contextual dimensions of how they perceived the barriers and facilitators for BSA women’s help-seeking shown in the adapted ecological intersectional model (Fig. 1). After that, codes were clustered into emerging themes through multiple reviews by all of the authors. Themes were clustered in each level (pre-determined) of the adapted ecological intersectional model, illustrated in Fig. 1 and known as the hybrid process of deductive and inductive theoretical thematic analysis [44]. NVivo 12 software was applied to facilitate data management, coding, and report generation [47, 48].

### Rigour

A number of strategies were applied to ensure rigour in this research. The target was to ensure that a wide range of service providers’ perspectives could be captured. All participant information sheets (PIS), consent forms, and interview guides were in multiple languages (English, Urdu, Hindi and Bengali). As interviews were remote, any eligible service provider in the UK was welcome to attend an interview. The researcher kept a reflexive journal, took field notes, and engaged in peer debriefing as a means of supporting and recording her reflections on the data as the process developed. The researcher has also reflected on her own thoughts, opinions and experiences to make the research process visible, which can help future researchers in ignoring “producing, reproducing, and circulating the discourse of research as a neat and linear process” [49]. To ensure trustworthiness and credibility, all authors met several times to discuss and compare the generated codes and themes to reconcile any discrepancies [50].

## Results

### Sample

The interviews involved 18 participants. All 18 participants—female DV service providers—gave verbal consent to take part. Most identified as South Asian (80%), while the remaining were Black or Arab (20%). To ensure confidentiality while tracing thematic patterns, coded identifiers (e.g., PROF1, PROF2) were used instead of pseudonyms.

As shown in Table 1 [51], the majority of participants were South Asian female service providers working in the third-sector/voluntary sector, all with direct experience supporting BSA women:

**Table 1:** Demographic overview of participants [51]

No.	ID	Ethnicity	Gender	Role	Experience with BSA Survivors	Organisational Sector
1	PROF 1	South Asian	Female	DV Service Provider	Yes	Third-sector/Voluntary
2	PROF 2	South Asian	Female	DV Service Provider	Yes	Third-sector/Voluntary
3	PROF 3	South Asian	Female	DV Service Provider	Yes	Third-sector/Voluntary
4	PROF 4	South Asian	Female	DV Service Provider	Yes	Third-sector/Voluntary
5	PROF 5	South Asian	Female	DV Service Provider	Yes	Third-sector/Voluntary
6	PROF 6	South Asian	Female	DV Service Provider	Yes	Third-sector/Voluntary
7	PROF 7	South Asian	Female	DV Service Provider	Yes	Third-sector/Voluntary
8	PROF 8	South Asian	Female	DV Service Provider	Yes	Third-sector/Voluntary
9	PROF 9	South Asian	Female	DV Service Provider	Yes	Third-sector/Voluntary
10	PROF 10	South Asian	Female	DV Service Provider	Yes	Third-sector/Voluntary
11	PROF 11	South Asian	Female	DV Service Provider	Yes	Third-sector/Voluntary
12	PROF 12	South Asian	Female	DV Service Provider	Yes	Third-sector/Voluntary
13	PROF 13	South Asian	Female	DV Service Provider	Yes	Third-sector/Voluntary
14	PROF 14	South Asian	Female	DV Service Provider	Yes	Third-sector/Voluntary
15	PROF 15	Black	Female	DV Service Provider	Yes	Third-sector/Voluntary
16	PROF 16	Black	Female	DV Service Provider	Yes	Third-sector/Voluntary
17	PROF 17	Arab	Female	DV Service Provider	Yes	Third-sector/Voluntary
18	PROF 18	Arab	Female	DV Service Provider	Yes	Third-sector/Voluntary

Service providers’ perceptions about barriers and facilitators for help-seeking behaviours among BSA women who have experienced DV were categorised according to the ecological intersectionality model [24]. Findings were structured using the ecological intersectionality model [24], mapping service providers’ perceptions of help-seeking barriers and facilitators across individual, relational, community, and structural levels. Participants’ professional experience supporting BSA DV survivors provided rich qualitative data. The synthesis of research data from the service provider interviews created an interconnection between the various levels of the ecological model with the barriers and facilitators for help-seeking behaviours among BSA women, men which result from their diverse intersectionality according to the ecological intersectionality model adapted from LaVoi [24].

### Stereotypical thinking and misuse of religious beliefs

This stereotypical thinking and misuse of religious beliefs belong at the community and relationship levels of the ecological intersectional model [24]. Service providers discussed stereotypical thinking, which is the set of beliefs and cognitive framework of traits, appearances, personalities, and behaviours of South Asian people as a group [52]. Participants shared about the role of some BSA women’s judgemental and biased thinking in normalising DV. They also informed that a number of intersectional features of ethnoreligious beliefs (honour, shame, victim blaming, patriarchal views, and misuse of religious beliefs) could be responsible for these stereotypical thoughts. Many participants felt that BSA women were very much embedded in their South Asian cultures and ethnoreligious groups, in which family honour and reputation plays an essential role in society, whereby they believe they would be humiliated for disclosing the abuse they suffer to others. It was also found that BSA women do not leave their homes even when they realise that they are experiencing abuse.

*“I think it’s very much rooted in – with the British South Asian women*,* it’s very much rooted in kind of fear of judgement*,* kind of. I think there’s a lot of shame and embarrassment there*,* shame*,* and feelings of guilt*,* like*,* I should not be experiencing this” (PROF13)*.

*“So the women have had children with the perpetrators and not realised that this is domestic violence and suddenly when they do realise*,* there’s another box of shame that all brings up as well” (PROF9)*.

*“.And again they also use religion*,* if you’re a religious person and you’ve had this warped understanding of religion*,* the men use it against the women that God will punish you if you don’t – if you’re not subservient to your husband” (PROF18)*.

Additional examples of patriarchal attitudes came from service providers’ interviews, with particular BSA women sharing that their previous mind-set had been to tolerate abuse, bearing all kinds of tortures, as they believed this was accepted in their cultures:

*“I think that the problem with the South Asian women is that they come*,* you know*,* we come from a mindset*,* a background*,* a culture where more than often it’s accepted*,* and I think it’s you know*,* they sort of – they’re too shy or too scared to talk about it. So a lot of women suffer from it*,* and they think it’s part of the norm*,* you know*,* it’s normal*,* it’s normal*,* and it should be we – they should have the patience to accept it*,* and tolerate it” (PROF5)*.

Participants informed that it is also necessary to understand the different South Asian cultures rather than viewing them all as one single category. For example, one participant stated:

*“You can’t treat all South Asian women the same—there are big differences between Bangladeshi*,* Indian*,* and Pakistani communities. What works for one might not work for another.”* (PROF7).

Moreover, they also emphasised not blaming the culture for DV (because, generally, DV may be present in all cultures, not specifically in South Asian culture).

*“…being really aware that South Asian communities is not just – you know*,* within South Asian*,* you do have different religions. You do have different practices. So it’s being mind – we use that terminology*,* culturally competent*,* so it’s being aware of how competent are you of someone else’s culture*,* but being mindful that that’s not an excuse for abuse” (PROF7)*.

### Fear of negative consequences

For BSA women, these fears were not only linked to personal safety, but also deeply shaped by cultural expectations surrounding honour, family reputation, and transgenerational impacts — such as concerns over siblings’ marriage prospects and immigration insecurity. This makes the experience of fear in this population distinct from general DV-related fears reported in other groups (9). The theme *“Fear of Negative Consequences”* encompasses a cluster of fears described by participants, including the potential for family rejection, financial hardship, damage to family honour, social exclusion, and immigration insecurity. These fears were often experienced as interconnected and central to the reluctance of BSA women to seek formal support. The professionals also responded to how BSA women, when disclosing abuse to seek help, would refuse to seek divorce or leave violent relationships due to the impact they think this would have on their siblings and their (the siblings’) futures, which could be hampered by their (the survivors’) marriage break-ups:

*“It’s very difficult because there’s so much stigma attached to domestic abuse culturally. You know*,* you’re bringing shame upon the family*,* the community*,* what are the community going to say*,* the impact it’s going to have on your children*,* your siblings. That’s a big one I came across*,* you know. If you’re the eldest or you’re the middle child*,* I mean*,* you know*,* how is it going to affect your brothers and sisters*,* you know*,* what are people going to say” (PROF3)*.

Some participants noted that women feared their divorce could damage their siblings’ reputations and marriage prospects, especially if they were the eldest.

This theme joins the relationship, community, and structural levels of the ecological intersectional model. Most service providers reported that fear is one of the strongest factors creating barriers to seeking help. Participants shared that, although support is available, women still cannot disclose abuse for fear of losing their families, fear of the perpetrator causing incidents with their families, fear of being blamed, and fear of facing shame. They also worried about their children’s futures, as some of them depend financially on the perpetrators, and are anxious about how society might accept them and their children if they are divorced, as this status seems to be taboo for some individuals in the South Asian community. Professionals shared that BSA survivors did not trust service providers for fear of them leaking their stories of abuse to the local South Asian communities. Participants’ voices are given below:

*“They also might fear that I’m Asian*,* they are Asian*,* and because there’s not a lot of trust within the Asian community*,* they fear that we might tell their families. And then*,* they’re also afraid as well about what might happen*,* or the threats that they’ve got. One of the threats is*,* if you tell anybody*,* watch we do to you. So*,* they’re afraid to tell us in case something happens or in case the family find out they’ve been getting support. So*,* there’s a lot of fear. A lot of fear” (PROF12)*.

*“I think it comes back down to the anxieties of where will I go*,* who will help me? I have no money. I have my children*,* you know*,* I have nothing. I’ll be on the streets” (PROF10)*.

Some providers, drawing on their own past experiences of DV, expressed empathy for survivors’ fears, having once faced similar emotional barriers themselves.

### Emotional state as barrier and facilitator

Among BSA women, emotional barriers to help-seeking were closely tied to cultural narratives of female endurance, internalised beliefs about preserving family cohesion, and religious interpretations of marriage obligations. Such factors contributed to emotional states—like guilt, shame, or denial—that were distinct from more general psychological impacts of DV seen in other contexts. Service providers noted several psychological consequences at the individual level of the ecological model. DV service providers informed that women do not share their experiences properly because of the emotional and psychological cost of remembering distressing events, which is extremely painful. BSA women often experience deep psychological distress, including numbness, self-blame, low confidence, and decision-making difficulties, which together form strong emotional barriers to seeking help. In their psychological trauma, BSA women also blame themselves for these incidents and continuously judge themselves, which are also risk factors in creating barriers to seeking help. They experience low self-esteem and low levels of self-confidence that prevent them from seeking help, either informally or formally. Service providers also talked about an unhealthy bond between perpetrators and some BSA women, whereby BSA women often realised from the beginning that they were suffering from abuse but disagreed that they were experiencing DV because they believed that their partner would be better if they (BSA women) would maintain kindly attitudes towards them. In contrast, some service providers described emotional readiness and resilience as key facilitators for help-seeking. Women who reached a turning point—such as exhaustion from long-term abuse, fear for their children’s safety, or a sense of self-worth—were more likely to seek formal support. One participant explained:

*“Sometimes it’s when they hit that emotional rock bottom—but with clarity. They say*,* ‘I can’t do this anymore*,* not for me*,* not for my kids.’ That’s when they’re ready to get help.”* (PROF12).

*“it was very difficult because they are accessing very painful memories*,* and*,* you know*,* that hurt was still very much there and they’re kind of working through that in therapy” (PROF13)*.

*“I guess*,* you know*,* thinking about the neuroscience*,* I mean*,* the brain isn’t going to be able to pick up things and connect things when they’re constantly in a threat and survival mode” (PROF17)*.

*“Others*,* they might have recognised it at the start*,* but they were very much in denial*,* or they were very much hoping that things would get better if they changed or if they were better themselves” (PROF13)*.

These findings suggest that while emotional barriers can be powerful inhibitors to help-seeking, shifts in emotional state—such as the emergence of hope, clarity, or strength—can also act as crucial facilitators.

### Informal help-seeking opportunities and barriers

Service providers described various informal help-seeking opportunities and barriers for BSA women which are connected with the relationship and individual levels of the ecological model. They reported that some BSA women are more likely to seek help from their families and relatives rather than from formal support services. However, while BSA women may be willing to seek help from people close to them, they may not manage to find the levels of support that they might expect from their families. Families may pressurise them to stay in the abusers’ homes as much as they can, even at the risk of death. Some families have also forcibly taken girls out of the UK to South Asian countries and forced them to be married to men from those countries. Participants informed that several BSA women are trapped by their families’ escalated adherence to social norms, which makes them feel bound to continue in these abusive relationships for their whole lives.

*“However*,* there’s still a lot of families that hold that kind of idea of like you know*,* you stay within your family*,* your relationship until you die type thing” (PROF8)*.

Some DV service providers have had DV experiences in their own lives, and shared how they had positively changed their lives by becoming involved in various activities. Most of the participants with previous experience of DV spoke of how their willingness to use self-help and self-disclosure to others as ways of seeking help were more effective than anything else.

*“So I was granted my divorce and then I think after divorce as done*,* I started up a childminding business*,* I continued with that. Then I started a cake business. And then I got into domestic abuse” (PROF18)*.

*“My second daughter ended up getting selective mutism*,* because she was so scared of him*,* she wouldn’t interact with men*,* and she wouldn’t talk to anybody but whisper only to me…I know all this ‘cos I’ve worked with children services as a domestic abuse specialist*,* so I realised afterwards the impact. And it took me another seven years to get my children in a good place*,* or a better place and then obviously I got married again and stuff” (PROF18)*.

Participants reported that a limited number of survivors had a close relationship with their friends and family, which healed them, as providing emotional support is effective for adapting to DV. It was also added that survivors felt free and comfortable in sharing their experiences with friends rather than with those in statutory roles.

*“Cos there was one lady that I spoke to who said for years she remained quiet because her sister-in-law was quite supportive*,* and she was kind of a shoulder to lean on. She wasn’t able to stop the violence altogether*,* but she was there as kind of a shoulder to lean on whenever she needed to talk or vent” (PROF15)*.

Participants consistently called for greater community engagement, recommending that families, neighbours, and religious leaders work to break stigma and create open dialogue. They proposed using local religious institutions and culturally relevant platforms to promote awareness and offer support.

### Formal help-seeking opportunities and barriers

Service providers shared a number of experiences from when they had provided support, while acting for DV organisations, to BSA women. These are aligned with the structural level in the ecological intersectional model. Participants shared thoughts about the barriers in reaching survivors because it is still the case that BSA women tend to keep their abuse secret. Moreover, service providers feel that there is a risk in talking about abuse in the community, and also that they would not pressurise BSA women to disclose DV. On the other hand, it was also helpful when the service providers were from a similar culture, religion and race/ethnic group, as the survivors would feel satisfied and comfortable with these service providers from their own (BSA women) background.

*“There was that cultural barrier*,* I think. So*,* I would say*,* yeah*,* just kind of more people from the same background*,* same culture*,* so that these women would feel comfortable” (PROF13)*.

*“At the moment it’s [Abuse] all very hush*,* hush*,* quiet*,* behind closed doors*,* and once we can – that is the biggest problem” (PROF5)*.

*“So*,* it’s really up to them. Our support is service user led*,* so it’s up to them if they want the support or not*,* we can’t force them to take that support” (PROF12)*.

Participants also reported the benefits of counselling, which helped BSA survivors to realise they were suffering from abuse and that this is a crime. They also mentioned that counsellors were not allowed to deliver any advice, but their positive encouragement and psychological therapy were effective in helping survivors to move forward. Participants informed that, compared with immigrant women with limited support for their temporary residence status, BSA women have more support available from every sector, if only they could be helped to recognise abuse, gain courage, and unfold their emotions. They also perceived that some BSA women had come to this realisation prior to counselling but they had not had the courage because they had been thinking about their children and families.

Counselling helped many BSA women recognise abuse and gain clarity. Although service providers noted they could not offer direct advice, their encouragement often empowered survivors. However, some women viewed therapy negatively due to cultural stigma around mental health.

The following participants also informed that some voluntary and statutory organisations expressed gender inequity and negative patriarchal views towards the survivors:

*Yes*,* I think there’s a stigma because there’s a lot of people who are non-white in organisations who don’t understand the patriarchal ideology that a lot of men have within this culture. And*,* you know*,* when I’ve had many women who have been interviewed by police or children’s services*,* and they don’t believe them (Survivors) for whatever reason (PROF18)*.

Several participants reflected on how their roles within third-sector or voluntary organisations made them more accessible to BSA women than statutory providers. They described being embedded in the community, offering services in familiar languages, and understanding the cultural contexts of clients’ decisions. This, they suggested, reduced stigma and made women more likely to approach them for help. One participant gave a quote:

*“I think they trust us more than the council or the police. We speak their language—not just literally*,* but emotionally and culturally. That makes a huge difference.”* (PROF10). Participants also noted that informal digital platforms, such as radio programmes and social media in women’s mother tongues, can play a key role in raising awareness and connecting BSA women with available support.

*“…you know*,* internet on the mobile or on the laptop or whatever PC they’ve got at home*,* that is the means of call… you know*,* a radio programme that they’ll be listening to*,* and they will have some advert saying*,* you know*,* don’t suffer in silence if you’re going through this*,* this*,* and that*,* then contact us. And it’s usually done in their mother tongue language*,* and that’s how they will understand that there is a service out there for them” (PROF11)*.

## Discussion

This research aims to identify the professionals’ perceptions of the barriers and facilitators for help-seeking behaviours among BSA women who have experienced DV. Previous studies have explored the challenges and facilitators for help-seeking behaviours primarily from the perspectives of DV survivors [20, 53–55]. In contrast, this study presents these issues from the viewpoints of service providers who support British South Asian women, offering additional insights based on their professional knowledge and frontline experience. Survivors often perceive statutory services as formal, mistrustful, or culturally insensitive 9, whereas voluntary sector providers describe greater cultural alignment, flexibility, and trust. This highlights the unique role of voluntary organisations in addressing barriers faced by BSA women. The overarching themes are: 1) stereotypical thinking and misuse of religious beliefs; 2) fear of negative consequences; 3) emotional disturbance as a barrier; 4) informal help-seeking opportunities and barriers; 5) formal help-seeking opportunities and barriers.

This research applies the ecological intersectional model to structure the barriers and facilitators across its multiple levels. These levels are shaped by the diverse, intersecting identities of BSA women, such as ethnicity, gender, religion, and racism. It is essential to recognise that these intersectional identarian factors contribute to diverse barriers and facilitators for help-seeking behaviours and that disregarding the identification of these intersectionalities leads to an incomplete understanding of the barriers and facilitators for help-seeking behaviours among these particular groups (BSA women). This is because these distinct identities can differentiate BSA women from other ethnic groups and the mainstream group, which will be helpful in securing support for this group (BSA women) [4, 23, 56]. These diverse, intersectional identities (e.g. ethnicity, gender, religion, and racism) have aligned with the four levels of the ecological model for discussing the various barriers and facilitators for help-seeking behaviours among BSA women.

The individual and relationship levels include BSA women’s psychological trauma as a noticeable barrier because DV has violated their ability to help themselves, and they find it hard to share or talk about DV with others. Similarly, some of the psychological theoretical literature has reported that women who have experienced DV have faced complications, called learned helplessness, in seeking support for this DV [57, 58]. Another comparable study confirmed that DV, in the form of psychological trauma caused by normal expectations and common ideas about the individual and the world, creates enormous confusion and unpredictability [59]. Consistent with the literature, this research has found similarities with a psychological phenomenon called ‘Stockholm Syndrome’, whereby some BSA women have lived with perpetrators without fleeing or seeking support because of their belief that the perpetrator would come right with their (BSA women’s) generosity [60]. The importance of self-help or self-disclosure as safety strategies has been stated, and at the individual level these are strong facilitators for help-seeking behaviours, with some articles sharing that some survivors have often stopped helping themselves and left everything to luck or god, which has prevented them from seeking support from others [61, 62]. Consequently, the authors of other studies have also emphasised how courage, self-confidence, and being open to self-care, in terms of healing, and to the gathering of more knowledge about healthy relationships can alert individuals to DV [63, 64]. BSA women have also found technology to be helpful in finding support at home or elsewhere. In accordance with such technology-related findings, previous studies have also shown the significance of seeking remote support [55], because technology-based research interviews or interventions can be accessed and self-managed from any location, and survivors can participate at any convenient time when the abusers are not at home [65–67]. Service providers also appreciate being able to deliver a remote service through digital systems, especially since this research has been conducted during the COVID-19 pandemic. These outcomes are consistent with other articles that have addressed the use of telephones/mobiles, social media and internet services in helping DV survivors [68–70].

At the relationship level, not only the partners or husbands, but the women’s in-laws and their own family members also play a vital role in developing barriers and limiting facilitators for seeking help. According to Abraham [71], the traditional power structure has been followed by some parents and in-laws (especially mothers-in-law) in some South Asian families whereby the women are treated as subordinate to the men. Through an intersectional lens, fear is one of the greatest barriers for South Asian women, which discourages women from seeking help or escaping DV situations [23]. For example, being of South Asian ethnicity and women of a specific religion, BSA women are afraid of facing friends, relatives, and the community with the stigmatisation of divorce, and of becoming isolated from their family and society, as well as continuously fearing about their children’s and siblings’ futures [72]. However, this current research also indicates the importance of family members, who could be the first contact of BSA women in seeking support, and the need for them to be well equipped, inspired and enabled to offer support to the BSA women [73]. The community level has some of the most significant barriers and facilitators, such as the patriarchal norms, the misuse of religious beliefs, and the treatment of abuse as a normal, private, or hidden matter within the community, all of which uphold South Asian family traditions. The findings support the evidence from previous observations of South Asian women trying to avoid unwanted negative social labelling [28]. Religion is one of the intersectional identities which is misused by perpetrators to justify wife-beating [63]. Perpetrators misuse religious beliefs in their abuse of BSA women and deliberately, but incorrectly, teach their wives (BSA women) that it is their responsibility to maintain relationships and also to keep DV a secret matter [50]. Although this study has found inadequate evidence of facilitators within the community for BSA women, most of the service providers have recommended building community support and awareness programmes to help BSA women to understand DV, to raise their voices, and to educate families and the community, because they might be in close contact with BSA women. Comparison of the findings with those of other studies confirms a more significant number of women who have experienced DV prefer to seek help from the people around them (parents, families, relatives, friends, and colleagues) instead of service providers or professional DV specialists [73, 74]. A novel contribution of this study is the insight into how professionals from third-sector and voluntary organisations perceive themselves as more culturally aligned and accessible to BSA women than statutory agencies. This distinction has been underexplored in previous research, which has largely focused on survivor perspectives or on formal/legal institutions. The findings here suggest that community-based providers can play a critical role in bridging cultural gaps and building trust in help-seeking pathways.

This study contributes to a rethinking of the label “hard to reach” often applied to British South Asian survivors of domestic violence. Service providers consistently described survivors as motivated to seek help, yet frequently blocked by entrenched cultural taboos, fear of community backlash, immigration concerns, and a lack of culturally safe services. These findings support the argument that it is not the women who are unreachable, but rather the services that are inadequately adapted to their needs (Cortis, 2011). Several participants from this study described cases where survivors felt judged or dismissed by statutory agencies, particularly during police interviews or child protection proceedings. In contrast, voluntary-sector organisations were seen as more flexible, community-embedded, and able to offer personalised, stigma-free support in native languages. This contrast often determined whether BSA women continued to seek help or retreated into silence. This reframing has critical implications for policy and practice, suggesting that service design must focus not only on outreach but also on restructuring support to be actively accessible, culturally competent, and trauma-informed.

These recommendations are comparable with other studies [75, 76], and such findings clearly indicate the need for novel community-based forms of support to address DV faced by BSA women who are in abusive relationships. The final level in the ecological model is the structural level, which includes the existence of barriers to formal help-seeking behaviours for DV, which are influenced by social inequalities [25] associated with formal support, such as limited funding for survivors, delayed provision of effective services, insufficient services after disclosure, lack of culturally competent education and awareness programmes, and lack of culturally appropriate services.

### Limitations

This study presents an integrated qualitative framework but does not apply formal network analysis techniques to quantify the relationships among the components. Future research could build on these findings by employing network analytic methods to further explore the complex interconnections identified here. Although there are diverse South Asian ethnic minority groups living in the UK, this study does not examine the DV experiences of women in any specific South Asian group, but has investigated the experiences of British South Asian women as a single category. The research participants (DV service providers) also provided information about all BSA women as a single group. However, the sensitive nature of the participants’ experiences, the COVID-19 pandemic and the government restrictions on social distancing across the whole country, as well as the limited time available within this PhD research, meant there were insufficient opportunities to re-interview or revisit participants. These remote interviews were also another limitation of this study relevant to other articles, as they limited the opportunity to reach more participants, and Internet connection problems sometimes caused inconvenience for interviewers and participants [77–80]. While this study employed a traditional researcher-led thematic analysis, future research could explore the use of AI-assisted approaches to enhance analytic transparency and efficiency. For example, Naeem, 2024 [81] outlines a structured process using ChatGPT to support theme development in qualitative research. Incorporating such tools—while maintaining critical reflexivity and human oversight—may offer valuable enhancements in large or multi-language datasets.

## Conclusions

### Practice and policy recommendations

This study aligns with the United Nations Sustainable Development Goals (SDGs), particularly Goal 5, which focuses on achieving gender equality and empowering all women and girls [82]. No studies have been conducted on the perceptions of service providers from third-sector domestic violence organisations about the help-seeking behaviours of British South Asian (BSA) women experiencing DV in the UK, although previous studies have found that the complexity of statutory services (the health sector, police, advocacy) can discourage women from seeking support [14, 28, 64]. There have also been several studies that have only discussed health professionals’ perceptions of DV [11, 12, 83], rather than those of other DV professionals. However, evidence from this current study has highlighted service providers’ perspectives on the basis of their practical experiences and training with respect to BSA women’s help-seeking behaviours in response to their experiences of DV. It is necessary to define the needs of BSA women who are experiencing DV while considering their ethnicity, culture, gender, and religion, as well as their intersectionality, in order to ensure there are appropriate facilitators that help them to seek support [76].

In this study, participants (service providers) have emphasised increasing awareness of DV at each level of the ecological model (individual, relationship, community, structural/social), as most of them have observed stereotypical thoughts (such as the isolation of divorced women, beliefs about women’s responsibilities to protect family honour by tolerating marital violence, obedience to patriarchal dominance, and victim-blaming) and a lack of awareness among BSA women about DV. They also highlighted the importance of counselling services, which have helped their clients (BSA women survivors) to recognise DV and regain positive strength to stop the abuse. Participants have recommended the creation of community and family awareness programmes involving BSA women, families, partners, community members, and religious leaders, because one of the major themes was BSA women’s lack of recognition of abuse. Such awareness programmes should be confidential and safe, and should follow careful cultural adaptations, because as the service providers noted, BSA women are concerned that service providers may not be trustworthy or may lack cultural competency. In this case, organisations should offer culturally appropriate specialist education and awareness programmes, as well as culturally appropriate services addressing both structural and cultural needs emerging from BSA women’s intersectional identities [75, 76, 82].

There is also a need to further develop remote services, such as online intervention programmes and events, DV awareness-related TV programmes, online advertisements of support information, and online/telephone/mobile helpline services. As this research was conducted remotely during the COVID-19 pandemic, it gathered information from participants about successful help-seeking strategies used by BSA women when they were isolated with perpetrators during the pandemic — consistent with findings in current research [83, 84].

## Conclusion

The findings of this study will help future researchers and service providers to carefully consider the intersectional identities of BSA women in developing culturally appropriate programmes and interventions. For example, BSA women with diverse gender, religious, and ethnic backgrounds should be included to minimise racism and to develop effective help-seeking strategies [82]. Policy should support BSA communities by ensuring culturally friendly approaches to social, health, and welfare services in both statutory and voluntary/third-party settings. It should also promote the development of culturally appropriate programmes and training for staff, professionals, and service providers to enhance their skills and understanding of the intersectionality of BSA communities. With proper education about South Asian cultural norms — grounded in cultural sensitivity — statutory and voluntary organisations will be better able to train service providers (including government domestic violence support staff, police, healthcare staff, social workers, family lawyers, and DV specialists from non-governmental organisations) to ensure survivors’ confidentiality, build trust, and offer comfort, safety, and security [30, 84].

This study makes an important contribution to SDG [76] 5: Gender Equality [82], by providing new insights into the barriers and facilitators for help-seeking among British South Asian women experiencing domestic violence. By foregrounding the perspectives of voluntary-sector service providers — a perspective underrepresented in previous research — the findings contribute to the development of culturally sensitive, trust-based service delivery models. These insights will help future practice, policy, and research to improve access to support for BSA women experiencing domestic violence.

## Data Availability

The datasets generated and/or analysed during the current study are not publicly available due to the sensitive nature of the data. This restriction is in place to protect the confidentiality and safety of the participants. But anonymous data is provided within the manuscript as quotes, Table 1.
